# The Microbiome-Mitochondria Axis in aging: a self-reinforcing vicious cycle linking metabolic dysregulation, mitochondrial quality control failure, and inflammaging

**DOI:** 10.3389/fmicb.2026.1874222

**Published:** 2026-07-22

**Authors:** Enshuo Liu, Jianbo Jia, Qi Liu, Chunyan Li, Shanhui Li, Tao Cai

**Affiliations:** 1 School of Sports Medicine and Health, Chengdu Sport University, Chengdu, China; 2 Gulin County Hospital of Traditional Chinese Medicine, Luzhou, China; 3 Sports Medicine Key Laboratory of Sichuan Province, Chengdu, China; 4 Affiliated Sports Hospital of Chengdu Sport University, Chengdu, China; 5 Institute of Sports Medicine and Health, Chengdu Sport University, Chengdu, China

**Keywords:** aging, inflammaging, metabolic dysregulation, Microbiome-Mitochondria Axis, mitochondrial quality control, mitophagy

## Abstract

Aging is a progressive degenerative process of cellular and systemic homeostasis in organisms, with mitochondrial dysfunction and altered intercellular communication as core hallmarks of this process. During aging, the gut microbiome and mitochondria exhibit a highly synchronized degenerative trajectory: this is characterized by decreased microbial diversity, reduced abundance of beneficial short-chain fatty acid (SCFA)-producing bacteria, and expansion of pro-inflammatory pathobionts in the gut, alongside impaired oxidative phosphorylation efficiency, excessive reactive oxygen species (ROS) production, and compromised quality control in mitochondria. Built on the evolutionary cornerstone of endosymbiotic theory, this review establishes a theoretical framework for the Microbiome-Mitochondria Axis (MMA) and proposes that the ancient molecular homology between mitochondria and modern gut bacteria has preserved a sensitive cross-species signal crosstalk mechanism. This review systematically dissects the bidirectional communication mechanisms of the MMA. First, microbial metabolites—including SCFAs, tryptophan-derived indole metabolites, and secondary bile acids—regulate mitochondrial energy metabolism, oxidative stress responses, and dynamic homeostasis via key signaling pathways such as AMPK-PGC-1α, AhR-Nrf2, and FXR/TGR5. Conversely, dysfunctional mitochondria actively reshape the gut microenvironment and propagate sterile inflammation through multiple pathways: mitochondrial ROS (mtROS)-mediated intestinal barrier disruption, metabolic reprogramming of immune cells toward a pro-inflammatory phenotype, and activation of the cGAS-STING innate immune pathway triggered by mitochondrial DNA (mtDNA) release. Here, we propose a unified theoretical framework centered on the MMA as a self-reinforcing pathological loop. In this model, gut dysbiosis drives depletion of beneficial microbial metabolites, which triggers mitochondrial quality control failure, mtDNA leakage, and inflammaging; in turn, inflammaging exacerbates gut dysbiosis by remodeling the intestinal microenvironment, thus forming a closed, self-amplifying vicious cycle. The MMA links multiple hallmarks of aging, including epigenetic alterations, immunosenescence, and stem cell exhaustion, providing a unifying pathological basis for age-related disorders such as neurodegenerative diseases, cardiovascular diseases, sarcopenia, and osteoarthritis. It also offers a systematic entry point for anti-aging interventions targeting the bidirectional metabolic-immune crosstalk between the microbiome and mitochondria.

## 1 Introduction

Aging is an inevitable, spontaneous process of progressive functional decline in organisms over time (López-Otín et al., 2023). In the evolution of aging research, the seminal framework of *The Hallmarks of Aging* proposed by López-Otín et al. has provided a systematic roadmap for dissecting organismal degenerative processes. Within this framework, mitochondrial dysfunction, as a core cellular hallmark, drives the cascade of aging-related pathologies (López-Otín et al., 2023), while altered intercellular communication offers a conceptual interface for host-microbiome interactions (Cox et al., 2026; Zachos et al., 2024). Gut microbial dysbiosis, an emerging systemic hallmark capable of reshaping the host’s metabolic and immune landscape, is increasingly becoming a central focus for understanding healthspan. However, most existing literature has focused on describing the phenotypic changes of the gut microbiome and mitochondria separately, with a lack of systematic dissection of the mechanisms underlying their deep functional coupling (Kulkarni and Gaikwad, 2025). However, the mechanistic link bridging gut-derived signals to mitochondrial responses remains inadequately defined.

In the process of unraveling the mechanisms of aging, the gut microbiome and mitochondria exhibit a highly synchronized degenerative evolutionary trajectory (Wen et al., 2025). At the systemic and macroscopic level, the gut microbiome displays distinct successional patterns with advancing age: decreased microbial diversity, reduced abundance of beneficial short-chain fatty acid (SCFA)-producing bacteria, and expansion of pro-inflammatory pathobionts, which collectively constitute a primary driver of inflammaging (Biagi et al., 2016). At the cellular and microscopic level, the decline of mitochondrial function is a defining hallmark of aging: impaired energy conversion efficiency, excessive ROS accumulation, dysregulated mitochondrial dynamics, and reduced mitophagy efficiency collectively characterize mitochondrial aging (Gray and Lang, 1999).

The cellular biological rationale for establishing the Microbiome-Mitochondria Axis (MMA) is first rooted in the evolutionary cornerstone of the Endosymbiotic Theory (Sagan, 1967): it posits that mitochondria originated from ancient prokaryotes, and this evolutionary homology has inherently preserved an extremely ancient and sensitive molecular crosstalk mechanism between mitochondria and modern gut microbes. Building on this evolutionary logic, this review aims to deepen and expand the connotation of the Microbiome-Mitochondria Axis in the field of aging research.

Unlike prior reviews that focused separately on mitochondria as a metabolic hub (Aguilar-López et al., 2020) or on the microbiome (Mallilankaraman et al., 2023), the present review specifically addresses the aging context and integrates both sides into a unified framework. More importantly, while existing studies have preliminarily explored gut-mitochondria crosstalk (Endres and Friedland, 2023), our model uniquely constructs a bidirectional feedback vicious cycle and highlights the active retrograde role of dysfunctional mitochondria in reshaping the gut ecosystem. This review thus establishes the MMA framework as a systematic basis for understanding the shared pathological mechanisms across multiple age-related diseases.

Critically, while previous reviews have acknowledged the existence of gut-mitochondria crosstalk, a systematic framework that conceptualizes this interaction as a closed-loop, self-reinforcing pathological cycle has been missing.

This review is based on a comprehensive literature search of PubMed, Web of Science, and Google Scholar databases up to April 2026, using keywords including “gut microbiota/microbiome,” “mitochondria,” “aging,” “inflammaging,” “mitochondrial quality control,” and “metabolic regulation.” A total of 147 relevant articles were critically evaluated and cited, covering both foundational mechanistic studies and recent advances in the field.

Building on this evidence base, this review fills the gap by (1) formally proposing the MMA vicious cycle model as a core driver of aging; (2) highlighting the active, retrograde role of dysfunctional mitochondria in perpetuating gut dysbiosis through mtDNA release and the cGAS-STING pathway; and (3) positioning the MMA as a unifying cellular hub that links multiple otherwise disparate hallmarks of aging, thereby reinforcing the cascade of metabolic dysregulation and mitochondrial quality control failure. In the following sections, we systematically construct this MMA vicious cycle and explore its far-reaching implications.

## 2 Core mechanisms by which the gut microbiome regulates mitochondrial function

Gut microbiota-derived metabolites regulate host mitochondrial function through at least four major categories of bioactive molecules—short-chain fatty acids (SCFAs), tryptophan-derived indole metabolites, secondary bile acids, and endotoxin (LPS). Based on 34 cited references, a unifying pattern emerges from this section: aging-associated depletion of beneficial metabolites (SCFAs, indole-3-propionic acid, secondary bile acids) and concurrent increase in endotoxin load synergistically impair mitochondrial bioenergetics, redox homeostasis, and quality control.

The gut microbiota participates in the regulation of host mitochondrial function through diverse metabolites and signaling molecules, serving as a critical bridge connecting environmental cues to cellular energy metabolism (Qiao et al., 2024). Microbe-derived metabolites enhance mitochondrial quality control, activate antioxidant pathways, and modulate fatty acid oxidation and amino acid metabolism, thereby shaping mitochondrial respiration and energy output (Moustakli et al., 2026). During aging, shifts in microbial community structure disrupt multiple metabolic signals, impairing mitochondrial energy production, redox homeostasis, and biogenetic capacity (Xiao et al., 2025; Luo et al., 2024) (Table 1).

**Table 1:** Core pathways of microbial metabolite-mediated regulation of mitochondrial function.

Metabolite class	Representative molecules	Primary receptors/pathways	Effects on mitochondrial function	Aging-associated changes	Key references
Short-chain fatty acids (SCFAs)	Acetate, propionate, butyrate	AMPK-PGC-1α	Promote biogenesis, enhance OXPHOS, maintain redox homeostasis	↓ SCFA-producing bacteria; ↓ SCFA levels	Hamed et al. (2023) and Li et al. (2022)
Tryptophan-indole metabolites	IPA, ILA, IAA	AhR-Nrf2, PXR	Enhance respiration, boost antioxidant defense, suppress inflammation	↓ IPA bioavailability	Feng et al. (2024), Han Z. et al. (2025), and Perdijk et al. (2024)
Secondary bile acids	DCA, LCA, T-β-MCA	FXR, TGR5	Regulate FAO, mitochondrial dynamics, and anti-inflammatory responses	Impaired microbiota-mediated biotransformation	Zhang J. et al. (2025), Velazquez-Villegas et al. (2018), and Zhang M. Y. et al. (2021)
Endotoxin	LPS	TLR4-NF-κB	Induce oxidative damage, mtDNA release, and inflammasome activation	↑ Translocation due to barrier dysfunction	Jiang et al. (2025), Hong et al. (2023), and Kalykaki et al. (2024)

### 2.1 Short-chain fatty acid-mediated regulation of mitochondrial energy metabolism

SCFAs—primarily acetate, propionate, and butyrate—are key fermentation products of dietary fiber (Hays et al., 2024). Butyrate plays a particularly prominent role in modulating mitochondrial function (Cavaliere et al., 2022): it directly enters the tricarboxylic acid (TCA) cycle to promote ATP synthesis (Dicks, 2024; Zhang M. et al., 2024) and activates AMPK-PGC-1α signaling to enhance mitochondrial biogenesis and oxidative phosphorylation (Hamed et al., 2023). Concurrently, SCFAs elevate NAD^+^ levels, improve electron transport chain efficiency, reduce ROS production, and preserve mitochondrial membrane potential (Li et al., 2022).

During aging, the gut microbiota undergoes profound dysbiosis, characterized by reduced microbial diversity and depletion of SCFA-producing bacteria (Araujo et al., 2024). Multiple studies confirm that fecal SCFA concentrations are significantly decreased in older adults, closely linked to altered gut microbial structure and diminished production of beneficial metabolites (Moustakli et al., 2026; Rees et al., 2025; Alqarni et al., 2026). As pivotal microbial metabolites, SCFAs not only fuel intestinal epithelial cells but also sustain mitochondrial function and redox homeostasis; their depletion weakens mitochondrial quality control and disturbs host energy metabolism (Zheng and Wang, 2021). Notably, SCFA deficiency is tightly associated with cellular senescence: SCFAs delay senescence by activating antioxidant pathways and suppressing the senescence-associated secretory phenotype (SASP) (Abavisani et al., 2025). When SCFA supply is insufficient, cellular energy metabolism is reprogrammed from efficient oxidative phosphorylation to inefficient glycolysis—a shift regarded as a core metabolic signature of cellular senescence, directly causing reduced ATP generation and functional decline (Araujo et al., 2024).

### 2.2 Regulatory effects of tryptophan metabolites on mitochondrial oxidative stress

The gut microbiota metabolizes dietary tryptophan via the indole pathway to generate a diverse array of bioactive metabolites, including indole-3-propionic acid (IPA), indole-3-lactic acid (ILA), and indole-3-acetic acid (IAA) (Jia et al., 2024; Su et al., 2022). Among these, IPA, a key microbial metabolite, is produced through biotransformation by bacteria such as *Clostridium* species (Ballanti et al., 2024). These microbiota-derived metabolites play critical roles in regulating mitochondrial function and redox homeostasis (Han Z. et al., 2025; Niu et al., 2025). In immune cells, IPA enhances mitochondrial respiratory function in CD4^+^ T cells by promoting fatty acid oxidation (FAO) and amino acid oxidation to boost energy metabolism (Li Q. et al., 2025). In the liver and intestine, IPA activates the pregnane X receptor (PXR) pathway to alleviate deoxynivalenol (DON)-induced oxidative stress and liver injury, while also regulating RNA methylation modifications (Feng et al., 2024). Meanwhile, IPA reduces mitochondrial superoxide anion production, inhibits abnormal mitochondrial respiration and reactive oxygen species (ROS) bursts in lung epithelial cells, and ameliorates inflammatory responses (Perdijk et al., 2024).

In parallel, indole metabolites activate the aryl hydrocarbon receptor (AhR) and Nrf2 antioxidant signaling pathways, while suppressing the NF-κB inflammatory pathway, thereby enhancing cellular antioxidant defense capacity (Gao X. et al., 2026). In addition to promoting mitochondrial respiration in CD4^+^ T cells and optimizing energy metabolism via enhanced FAO and amino acid oxidation (Li Q. et al., 2025), IPA upregulates mitochondrial transcription factor A (Tfam) in bone tissue to support mitochondrial biogenesis and function (Xu et al., 2025). Multi-omics studies further indicate its synergistic effects with pathways such as PPARα (Zhang Y. et al., 2025). IPA supplementation has demonstrated broad protective effects across multiple age-related disease models, including improved musculoskeletal health, extended lifespan in Drosophila, attenuated myocardial fibrosis, and preserved blood–brain barrier integrity (Vyavahare et al., 2026; Lu et al., 2025; Wang Y. C. et al., 2024; Pan C. et al., 2025). Collectively, the tryptophan-indole metabolic axis emerges as a key regulatory hub for microbiota-host mitochondrial crosstalk, which plays a central role in maintaining redox homeostasis, inhibiting inflammation, and delaying age-related pathological processes. Furthermore, reduced IPA levels resulting from gut dysbiosis (e.g., periodontitis, antibiotic exposure) are closely linked to metabolic dysfunction-associated steatotic liver disease (MASLD), neuroinflammation, and mitochondrial dysfunction (Ren et al., 2025).

### 2.3 Bile acid metabolism and the regulation of mitochondrial energy homeostasis

The gut microbiota acts as a critical “biotransformer” in host bile acid metabolism. Primary bile acids synthesized in the liver enter the intestinal tract, where they are converted into secondary bile acids such as deoxycholic acid (DCA) and lithocholic acid (LCA) by bacterial bile salt hydrolase (BSH) and related reductases (Han L. et al., 2025; He et al., 2024). This biotransformation process not only alters the physicochemical properties of bile acids, but more importantly, reshapes their endocrine function as signaling molecules, thereby deeply participating in the regulation of mitochondrial energy metabolism (He et al., 2026).

As key signaling molecules, secondary bile acids regulate host metabolism and mitochondrial function primarily through two pathways: the nuclear receptor farnesoid X receptor (FXR) and the membrane receptor G protein-coupled bile acid receptor 1 (GPBAR1, commonly known as TGR5). FXR activation modulates the expression of genes related to lipid and energy metabolism, for example, by upregulating the expression of key enzymes in fatty acid oxidation such as carnitine palmitoyltransferase 1α (CPT1α), to enhance mitochondrial fatty acid utilization capacity (Zhang J. et al., 2025). TGR5 activation, in turn, participates in energy homeostasis regulation and anti-inflammatory responses through non-genomic signaling pathways such as cAMP/PKA (Rosatelli et al., 2023; Darmanto et al., 2025). The two receptors act synergistically to inhibit the NF-κB pathway and pro-inflammatory polarization of macrophages, reduce the release of inflammatory factors such as TNF-*α* and IL-1β, and indirectly alleviate inflammation-induced mitochondrial oxidative damage (Thibaut and Bindels, 2022; Biagioli et al., 2023). The gut microbiota regulates bile acid deconjugation and biotransformation via BSH and the bai gene cluster, modulating the production of secondary bile acids including tauro-*β*-muricholic acid (T-β-MCA, an FXR inhibitor) and LCA (a TGR5 agonist), and thereby tuning FXR/TGR5 signaling activity (Gao G. et al., 2026). Gut dysbiosis (e.g., reduced abundance of BSH-producing bacteria) can lead to disrupted bile acid profiles and impaired receptor activation, weakening its regulatory effects on mitochondrial function and inflammation (Huang et al., 2022; Wang B. et al., 2024). Notably, interventions such as 5-aminosalicylic acid (5-ASA) ameliorate bile acid metabolism and colitis by modulating FXR/TGR5 signaling only when the gut microbiota is intact, and this protective effect is abolished following microbiota depletion, highlighting the essential role of the gut microbiota in this axis (Huang et al., 2022). Furthermore, FXR deficiency or signaling inhibition is closely associated with the progression of metabolic liver disease and age-related liver injury (Yang et al., 2023; Wooton-Kee et al., 2025).

Taken together, the bile acid-microbiota-FXR/TGR5 axis constitutes a core hub linking the gut microenvironment to mitochondrial energy homeostasis and inflammatory regulation, and its functional decline participates in the vicious cycle of “metabolic dysfunction-mitochondrial damage-chronic inflammation” (Zhang R. et al., 2024; Shi et al., 2026). At the organelle dynamics level, the TGR5 signaling pathway directly regulates the dynamic balance of mitochondrial fission and fusion. Studies by Velazquez-Villegas et al. (2018) demonstrated that TGR5 activation induces mitochondrial fission via the ERK-DRP1 axis, enhancing mitochondrial respiratory function in adipocytes. Meanwhile, as a Gs protein-coupled receptor, TGR5 activation increases intracellular cAMP levels (Kawamata et al., 2003) and activates PKA, which in turn inhibits the GTPase activity and mitochondrial translocation of Drp1 by phosphorylating its Ser637 residue, thereby antagonizing excessive mitochondrial fission and promoting elongation of the mitochondrial network (Chang and Blackstone, 2007). Recent studies further confirmed that TGR5 signaling inhibits mitochondrial fission and enhances PINK1/Parkin-mediated mitophagy via regulating the PKCδ/Drp1 pathway (Zhang M. Y. et al., 2021). In addition, bile acids (e.g., DCA and cholic acid [CA]) induce mitochondrial dysfunction and impaired autophagic flux in skeletal muscle cells, and their sarcopenic effects have been confirmed to be TGR5-dependent (Abrigo et al., 2023). Therefore, the secondary bile acid-TGR5 signaling axis exerts bidirectional regulatory effects on mitochondrial dynamics, and the decline of this fine-tuning mechanism may act as an upstream driver of mitochondrial fragmentation and failure of the mitochondrial quality control system during aging.

### 2.4 Mechanisms of endotoxin-mediated mitochondrial inflammatory injury

In pathological conditions with impaired intestinal barrier integrity, gut dysbiosis can disrupt intestinal epithelial barrier function, promoting the translocation of lipopolysaccharide (LPS), a cell wall component of Gram-negative bacteria, into the bloodstream. This results in metabolic endotoxemia, a key link between gut microbial dysregulation and systemic chronic inflammation (Kalyan et al., 2022; AlAbduljader et al., 2025). The mechanisms underlying barrier dysfunction involve mitochondrial abnormalities in intestinal epithelial cells (IECs). IEC mitochondria actively regulate the maintenance of gut microbial homeostasis, intestinal metabolism, and the immune barrier; insufficient energy supply from these mitochondria impairs barrier repair capacity, forming a bidirectional dependent relationship between mitochondria and barrier function (Wang et al., 2023). Meanwhile, gut dysbiosis leads to reduced microbial diversity and decreased butyrate-producing bacteria, further compromising barrier integrity. On one hand, this promotes the translocation of harmful molecules such as LPS into the circulation, and on the other hand, it is accompanied by a reduction in SCFA-producing bacteria and decreased intraluminal SCFA concentrations (Zhu et al., 2026; Ashiqueali et al., 2025).

LPS activates TLR4 signaling, driving pro-inflammatory cytokine release (TNF-*α*, IL-6) and chronic low-grade inflammation (Jiang et al., 2025). Concurrently, LPS suppresses electron transport chain activity, elevates ROS production, and compromises ATP synthesis (Hong et al., 2023; Shikuma et al., 2022). Damaged mitochondria release cell-free mtDNA, which acts as a damage-associated molecular pattern (DAMP) to further amplify inflammatory signals via pathways including cGAS-STING (Picca et al., 2022). Meanwhile, mitochondrial ROS (mtROS) is a key trigger for NLRP3 inflammasome activation, promoting caspase-1 activation and the maturation and release of IL-1β and IL-18, creating an inflammatory cascade amplification effect (Kalykaki et al., 2024). In the context of aging, this pathological axis exhibits “dual vulnerability”: on one hand, age-related gut dysbiosis exacerbates barrier damage and endotoxin translocation (Aldriwesh et al., 2026; Wang J. et al., 2025); on the other hand, age-associated declines in mitochondrial antioxidant capacity, impaired autophagic clearance, and the state of inflammaging render mitochondria more susceptible to attack by LPS and inflammatory factors, leading to the continuous accumulation of functional damage (Kalykaki et al., 2024; Du et al., 2025). This gut-mitochondria-inflammation axis has been implicated in metabolic, neurodegenerative, and bone disorders (Ju et al., 2026; Cai et al., 2024), and the LPS-TLR4 pathway represents a promising therapeutic target (Hong et al., 2023).

**Figure 1:**
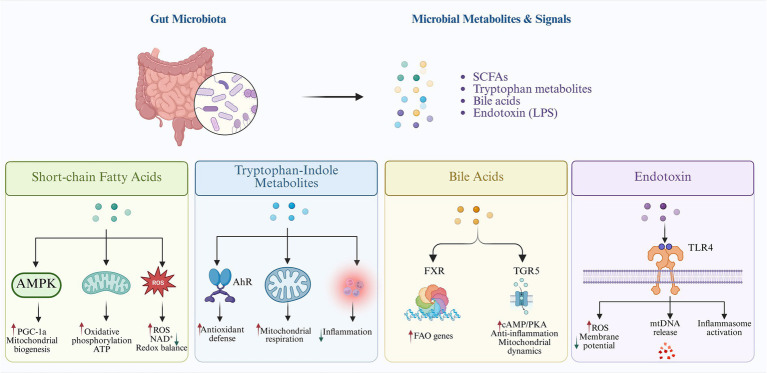
Core mechanisms by which gut microbiota regulates mitochondrial function via microbial metabolites.

**Figure 2:**
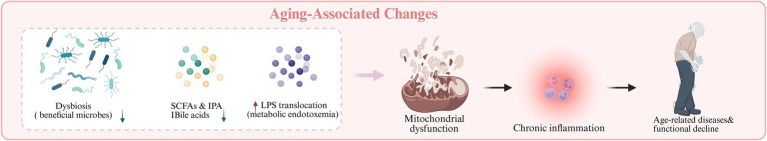
Aging-associated dysregulation of the gut microbiota-mitochondria regulatory network.

In summary, the gut microbiota synergistically regulates mitochondrial function at multiple levels, including energy metabolism, redox homeostasis, and inflammatory regulation, through diverse metabolic signals such as short-chain fatty acids, tryptophan-derived indole metabolites, and bile acids. In contrast, endotoxin (LPS) amplifies inflammatory responses and exacerbates mitochondrial damage in the setting of gut dysbiosis. These pathways intersect with one another to collectively maintain the dynamic balance between metabolism, mitochondria, and immunity. During aging, with the reduction of beneficial metabolites and increased endotoxin load, this regulatory network is progressively destabilized, driving mitochondrial functional decline and the progression of chronic inflammation (Figures 1, 2).

The gut microbiota produces diverse metabolites, including Short-chain fatty acids (SCFAs), Tryptophan-indole metabolites, Bile acids, and Endotoxin (lipopolysaccharide, LPS). SCFAs activate the AMPK pathway, promoting mitochondrial biogenesis via PGC-1α, enhancing oxidative phosphorylation, and maintaining redox balance. Tryptophan-indole metabolites (e.g., IPA) activate AhR to enhance antioxidant defense and mitochondrial respiration while suppressing inflammation. Bile acids regulate fatty acid oxidation (FAO) genes via FXR and promote anti-inflammatory responses and mitochondrial dynamics via TGR5/cAMP/PKA signaling. Endotoxin (LPS) binds TLR4 on the cell surface, inducing oxidative stress, mtDNA release, and inflammasome activation, thereby contributing to mitochondrial dysfunction.

During aging, gut dysbiosis leads to a decrease in beneficial metabolites (SCFAs, IPA, bile acids) and an increase in LPS translocation (metabolic endotoxemia). This environmental shift drives mitochondrial dysfunction, triggers chronic low-grade inflammation (inflammaging), and ultimately leads to age-related diseases and functional decline of the body.

## 3 Mechanisms underlying the retroactive regulation of the gut microenvironment by mitochondria

Mitochondria not only respond to microbial signals but also actively reshape the gut microenvironment through retrograde signaling. Reviewing 28 references, this section identifies four such pathways: (1) excessive mitochondrial ROS (mtROS) production disrupts intestinal barrier integrity; (2) mitochondrial-driven metabolic reprogramming polarizes immune cells toward a pro-inflammatory phenotype; (3) mitochondrial DNA (mtDNA) release activates cGAS-STING and NLRP3 inflammatory cascades; and (4) the mitochondrial unfolded protein response (UPRmt) triggers mitokine release (FGF21, GDF15) that increases intestinal permeability. The common theme is the transition from homeostatic maintenance to positive feedback amplification of inflammation and dysbiosis during aging.

As core organelles regulating cellular energy metabolism, calcium homeostasis, and oxidative stress responses, mitochondria not only maintain moderate levels of reactive oxygen species (ROS) to support signal transduction and cellular homeostasis, but also deeply integrate innate immune responses by modulating the energy metabolism and phenotypic functions of immune cells (Qiao et al., 2024; Li Q. et al., 2025) (Table 2). During aging, mitochondrial function declines markedly, characterized by dysregulated biogenesis and dynamics, accumulation of mtDNA mutations, reduced ADP sensitivity, and exacerbated oxidative stress (Ren et al., 2022; Yang L. et al., 2024), which in turn trigger aberrant metabolic reprogramming and the release of damage-associated molecular patterns (DAMPs).

**Table 2:** Mitochondrial retrograde pathways reshaping the gut microenvironment.

Pathway	Key mediators	Effects on gut microenvironment	Aging-associated amplification	Key references
Excessive mtROS production	Reactive oxygen species	Disrupt tight junctions, ↑ permeability, promote LPS translocation	Impaired antioxidant capacity; ↓ ROS clearance	Zhang C. et al. (2022), Deng et al. (2022), Chen et al. (2024), Zhao et al. (2025), and Odnokoz et al. (2023)
Immune metabolic reprogramming	HIF-1α, NF-κB	Drive M1 polarization, release pro-inflammatory cytokines, remodel microbiota	QC failure → persistent glycolytic state	Zhong et al. (2025), Yang et al. (2026), Sun et al. 2022, Zhang Y. et al. (2022), Liu C. et al. (2026), Li X. et al. (2025), Morris et al. (2022), Pan et al. (2022), Wang et al. (2026), and Guo and Shao (2025)
mtDNA release & innate immune activation	cGAS-STING, TLR9, NLRP3	Amplify inflammatory cascades via type I IFNs and pro-inflammatory cytokines	Sustained mtDNA leakage → chronic inflammation	Pan P. et al. (2025), Lauf et al. (2024), Irazoki et al. (2023), Xian et al. (2022), and Zhao et al. (2021)
UPRmt & mitokine signaling	FGF21, GDF15	Downregulate tight junction proteins, ↑ permeability, inhibit AMP expression	Chronic UPRmt activation → persistent barrier damage	Urbauer et al. (2024) and Ruiz-Malagón et al. (2025)

### 3.1 Mitochondrial ROS-driven imbalance of intestinal barrier function and microbiota remodeling

Mitochondria-derived reactive oxygen species (mtROS) play a dual role in the homeostasis and damage of the intestinal barrier. Under pathological conditions, mitochondrial dysfunction leads to excessive mtROS production, which directly damages the intestinal epithelial barrier. In addition, deoxynivalenol (DON) exposure causes loss of mitochondrial membrane potential, opening of permeability transition pores, and significant downregulation of the tight junction proteins Occludin, ZO-1, Claudin-1/3, and N-Cad, along with increased lactate dehydrogenase (LDH) release, indicating impaired barrier integrity (Zhang C. et al., 2022). Similarly, mycophenolic acid (MPA) has been confirmed to induce downregulation of tight junction protein expression and barrier integrity impairment in Caco-2 cells through triggering mitochondrial dysfunction and subsequent oxidative stress (Deng et al., 2022). Notably, MPA-induced mitochondrial damage exhibits a concentration-dependent pattern: at low concentrations, it induces mitochondrial membrane potential hyperpolarization and cytochrome c accumulation within mitochondria, while at high concentrations, it causes collapse of the mitochondrial membrane potential and cytochrome c release. Excess ROS not only oxidatively modifies tight junction structures, but also disrupts the mucus layer, damages goblet cells, impairs mechanical and chemical barriers, and disturbs the intestinal oxygen gradient and microenvironment, indirectly triggering shifts in bacterial niches and gut dysbiosis (Chen et al., 2024; Zhao et al., 2025).

Increased barrier permeability promotes the translocation of intraluminal microbes and their metabolites, activating inflammatory pathways such as TLR4-NF-κB/MAPK and releasing pro-inflammatory factors including TNF-*α* and IL-6, which further exacerbate oxidative stress and mitochondrial damage (Liu et al., 2023; Liu Y. et al., 2026). Consequent disruptions of mitochondrial homeostasis, caused by reduced expression of mitochondrial fusion proteins, STARD7 deficiency, or Hsp60 knockout, are all closely associated with abnormal distribution of tight junction proteins, increased intestinal permeability, and chronic inflammation (Urbauer et al., 2024; Uddin et al., 2024). Notably, multiple intervention strategies exert protective effects by targeting this core axis: for example, nanozymes scavenge ROS and upregulate ZO-1/Occludin expression (Yang Y. et al., 2024), selenium nanoparticles (SeNPs) improve barrier function by regulating mitochondrial function and the gut microbiota (Qiao et al., 2022), and KLB protein protects against alcohol-induced injury by stabilizing TRPV6 and tight junction complexes (Hou et al., 2022). Furthermore, loss of mitochondrial Prx activity leads to barrier dysfunction associated with systemic inflammation and a progeroid phenotype (Odnokoz et al., 2023). Critically, the terminal differentiation of goblet cells and mucin secretion are highly dependent on energy supply from mitochondrial oxidative phosphorylation, and impaired mitochondrial ATP synthesis can directly compromise the physical isolation function of the mucus barrier (Sünderhauf et al., 2021).

### 3.2 Mitochondrial metabolic reprogramming regulates immune cell polarization and the intestinal inflammatory microenvironment

As the core of cellular energy metabolism and a key hub of immune regulation, the functional state of mitochondria directly determines the phenotypic differentiation and functional orientation of immune cells. Under homeostasis, macrophages utilize OXPHOS for energy, supporting anti-inflammatory M2 polarization and intestinal immune tolerance (Zhong et al., 2025; Yang et al., 2026; Sun et al., 2022). Dysregulation of mitochondria-immune crosstalk is a key driver of intestinal inflammation (Zhang Y. et al., 2022). Aging or chronic inflammation impairs mitochondrial function, elevates mtROS, and drives metabolic reprogramming toward glycolysis. This shift, mediated by HIF-1*α* stabilization and NF-κB activation, promotes M1 polarization and pro-inflammatory cytokine release (Li X. et al., 2025; Morris et al., 2022). Microbiota metabolites such as SCFAs modulate this balance through epigenetic mechanisms (Liu C. et al., 2026), but this regulation is progressively lost during aging.

M1-type cells secrete large amounts of TNF-α, IL-6, IL-1β, and nitric oxide, which disrupt intestinal epithelial tight junctions, induce apoptosis, impair intestinal barrier integrity, and alter the local redox microenvironment (Pan et al., 2022). This change exerts a strong selective pressure on the gut microbiota: anaerobic bacteria are suppressed, while aerotolerant or facultative anaerobic bacteria expand, leading to a shift in the microbial community toward a pro-inflammatory profile; increased endotoxin load further amplifies inflammatory signals, forming a vicious cycle of “metabolic reprogramming-enhanced inflammation- microbiota dysbiosis” (Wang et al., 2026; Guo and Shao, 2025).

### 3.3 Mitochondrial DNA release-mediated innate immune amplification and intestinal ecological disruption

Under conditions of mitochondrial dysfunction or sustained oxidative stress, mitochondrial DNA (mtDNA) can be aberrantly released into the cytoplasm or extracellular space through multiple mechanisms, including mitochondrial permeability transition pore (mPTP) opening, gasdermin-mediated pore formation, and mitochondria-derived vesicle trafficking (Yang Y. et al., 2025; Zhao et al., 2023; Li et al., 2023). Accumulated mtROS not only damages mtDNA but also inhibits mitophagy, leading to sustained mtDNA release (Wang M. et al., 2024; Zhou H. et al., 2025). Due to its unmethylated CpG-rich structure and lack of histone protection, mtDNA is highly immunogenic and susceptible to recognition by intracellular DNA sensors (Algieri et al., 2025; Xia et al., 2025).

Released mtDNA triggers inflammation via three core pathways: (1) cytosolic cGAS-cGAMP-STING-TBK1-IRF3 axis inducing type I interferons (Pan P. et al., 2025; Lauf et al., 2024); (2) endosomal TLR9-MyD88-NF-κB signaling promoting TNF-*α* and IL-6 release (Irazoki et al., 2023); and (3) oxidized mtDNA directly activating the NLRP3 inflammasome (Xian et al., 2022). These pathways synergistically amplify inflammatory cascades, a mechanism confirmed in multiple pathological contexts including intestinal injury, diabetic cardiomyopathy, and Alzheimer’s disease (Song et al., 2026; Yan et al., 2022; Akhter et al., 2025).

mtDNA-driven immune activation can form a positive feedback cycle of “damage – inflammation – secondary damage,” which is significantly exacerbated in the context of aging. For example, aberrant STING expression in aged macrophages impairs interferon regulatory capacity (Lauf et al., 2024), and the decline of mitochondrial quality control leads to sustained mtDNA leakage, driving chronic low-grade inflammation (inflammaging) (Zhao et al., 2021). At the tissue level, this pathway is involved in the disruption of the intestinal epithelial barrier (Song et al., 2026), corneal epithelial homeostasis imbalance (Fan et al., 2024), the progression of cardiac hypertrophy (Zhou H. et al., 2025), and neurodegenerative diseases (Zhao et al., 2021). Notably, endotoxins such as LPS produced by dysbiotic microbiota can directly exacerbate mitochondrial damage and induce mtDNA release into the cytoplasm, thereby driving inflammatory cytokine secretion and pyroptosis, forming a pathological amplification network across organelles, the immune system, and the microenvironment (Zhang Y. F. et al., 2021). Targeted regulation of mtDNA release (e.g., inhibiting VDAC1 or enhancing Mfn2 expression) or blocking the cGAS-STING pathway has emerged as a potential strategy for the intervention of multiple inflammatory diseases (Lang et al., 2025).

In summary, mitochondria retroactively shape the structure of the gut microenvironment through multiple pathways, including ROS production, metabolic reprogramming, and mtDNA release, at the levels of intestinal barrier homeostasis, immune cell function, and innate immune signal amplification. Among these, excessive mtROS and mtDNA-mediated inflammatory responses can disrupt the barrier and drive a pro-inflammatory shift in the microbiota, while the metabolic state of mitochondria determines the direction of immune cell polarization, further shaping the intestinal inflammatory microenvironment.

### 3.4 The mitochondrial unfolded protein response and long-range communication with the intestinal microenvironment

In addition to ROS-induced damage and mtDNA leakage, mitochondrial dysfunction also activates an adaptive stress signaling pathway known as the mitochondrial unfolded protein response (UPRmt). In the intestinal epithelium, the UPRmt not only regulates mitochondrial protein homeostasis within the cell itself, but also achieves long-range regulation of the microenvironment through the secretion of signaling molecules termed mitokines (Urbauer et al., 2024).

Mitokines are secretory factors induced by mitochondrial stress, mainly including fibroblast growth factor 21 (FGF21) and growth differentiation factor 15 (GDF15). In the colonic mucosa of patients with inflammatory bowel disease (IBD), sustained UPRmt activation is accompanied by a significant increase in GDF15, which directly increases intestinal barrier permeability by downregulating the tight junction proteins ZO-1 and claudin-1 (Ruiz-Malagón et al., 2025). In addition, mitochondrial stress can indirectly affect intestinal epithelial renewal and the expression of antimicrobial peptides (AMPs) by regulating the Wnt signaling pathway, thereby reshaping the colonization resistance of the microbiota (Urbauer et al., 2020); the evolutionarily conserved UPRmt-Wnt communication axis has been confirmed in model organisms to mediate long-range intestinal mitochondrial surveillance (Liu et al., 2024).

This UPRmt-mediated microenvironmental remodeling can produce significant feedback effects at the microbiota level. Through intestinal epithelial-specific knockout of the mitochondrial chaperone protein Hsp60, Urbauer et al. found that mitochondrial perturbation does not cause colonic damage in germ-free mice, but induces the expansion of *Bacteroides* and IBD-like pathological changes in the presence of a commensal microbiota (Urbauer et al., 2024). This directly demonstrates that mitochondrial dysfunction can systematically alter the intestinal microenvironment via UPRmt signaling, thereby selectively reshaping the microbial composition.

**Figure 3:**
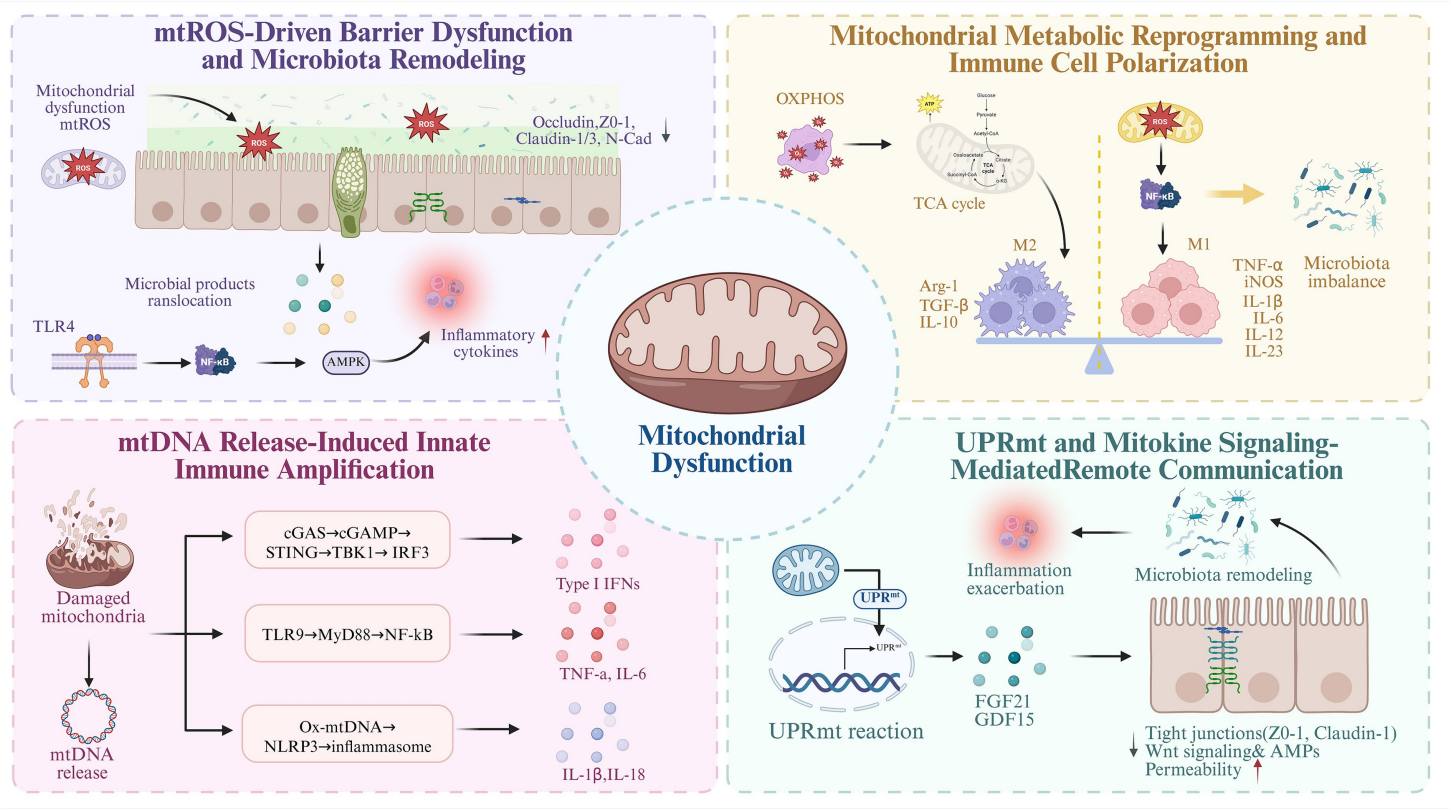
Core retroactive mechanisms of mitochondrial dysfunction on the gut microenvironment.

Taken together, the UPRmt and its mediated mitokine signaling constitute another important branch of the retroactive regulation of the gut microbiota by mitochondria, acting synergistically with ROS-induced damage and the mtDNA/cGAS-STING inflammatory pathway to jointly drive the progression of gut microecological imbalance and sustained chronic low-grade inflammation (Figure 3).

It is important to note that the UPRmt is not exclusively a pathological effector. In its early or moderate phases, the UPRmt serves as an adaptive, protective feedback mechanism—a phenomenon termed mitohormesis—that enhances mitochondrial proteostasis and cellular resilience against stress. This negative feedback arm likely predominates during most of the lifespan. However, with advancing age and the accumulation of persistent mitochondrial damage, this protective response can become overwhelmed and maladaptive, as evidenced by the chronic elevation of mitokines such as GDF15, which then paradoxically contributes to barrier dysfunction and sustained inflammation. This shift from a protective to a pathological UPRmt signature may represent a critical tipping point in the transition from homeostatic aging to the self-reinforcing MMA vicious cycle.

Mitochondrial dysfunction reshapes the gut ecosystem through four primary pathways: ① Excessive mtROS disrupts intestinal epithelial tight junctions (e.g., Occludin, ZO-1, Claudin-1/3), increases intestinal permeability, and promotes microbial product translocation, activating TLR4/AMPK and inflammatory pathways. ② Metabolic reprogramming of immune cells from OXPHOS to glycolysis drives macrophage polarization toward a pro-inflammatory M1 phenotype, releasing pro-inflammatory cytokines and exacerbating microbiota dysbiosis. ③ mtDNA leakage into the cytosol activates innate immunity via three major pathways: cGAS-STING, TLR9, and NLRP3 inflammasome, amplifying the inflammatory cascade. ④ Activation of the mitochondrial unfolded protein response (UPRmt) triggers the release of mitokines including FGF21 and GDF15, which increase intestinal permeability by downregulating tight junction proteins and inhibiting antimicrobial peptide (AMP) expression, thereby remodeling the gut microbiota.

## 4 Systemic vicious cycle of aging driven by metabolic dysregulation and mitochondrial quality control failure

### 4.1 Positive feedback closed loop at the cellular level

Having delineated the forward (microbiome-to-mitochondria) and retrograde (mitochondria-to-microbiome) pathways in Sections 2 and 3, this section integrates these bidirectional lines of evidence to formally construct the unified Microbiome-Mitochondria Axis (MMA) vicious cycle model. The core synthesized pattern, supported by 18 key references, is that depletion of beneficial microbial metabolites and mitochondrial dysfunction form a closed-loop system operating through metabolic-immune crosstalk at both cellular and systemic levels—a self-amplifying cycle that drives inflammaging and connects multiple hallmarks of aging.

Based on the positive regulatory mechanisms of gut microbial metabolites on mitochondrial quality control elucidated in Chapter 2, and the retroactive remodeling effects of dysfunctional mitochondria on the gut microenvironment dissected in Chapter 3, this section constructs and establishes the vicious cycle of Microbiome-Mitochondria Axis (MMA) dysregulation as the core theoretical framework of this review. At the cellular level, this self-reinforcing cascade process can be described in pathological order as follows:

Initial age-related or environmental factors induce gut dysbiosis, with a reduction in short-chain fatty acid-producing bacteria and expansion of pro-inflammatory pathobionts (Araujo et al., 2024), resulting in a significant decrease in the bioavailability of beneficial microbial metabolites, including butyrate, indole-3-propionic acid (IPA), and secondary bile acids (Moustakli et al., 2026; Rees et al., 2025; Jia et al., 2024). Metabolite depletion directly impairs the host mitochondrial quality control system, covering key processes such as mitochondrial biogenesis, dynamic homeostasis, and mitophagy (Hamed et al., 2023; Li et al., 2022), triggering reduced oxidative phosphorylation efficiency, excessive mitochondrial reactive oxygen species (mtROS) production, and impaired mitochondrial DNA (mtDNA) integrity (Ren et al., 2022; Yang L. et al., 2024).

Dysfunctional mitochondria reshape the microenvironment through three temporally and functionally interconnected pathways. First, excessive mtROS disrupts intestinal tight junctions, increases permeability, and promotes bacterial/endotoxin translocation (Zhang C. et al., 2022; Deng et al., 2022; Chen et al., 2024; Zhao et al., 2025), creating an initial inflammatory trigger. Second, mitochondrial dysfunction drives immune metabolic reprogramming from OXPHOS to glycolysis, promoting M1 macrophage polarization and pro-inflammatory cytokine release (TNF-*α*, IL-6), which further damages the barrier and alters the redox microenvironment, selectively expanding facultative anaerobic pathobionts (Zhong et al., 2025; Yang et al., 2026; Sun et al., 2022; Liu C. et al., 2026; Li X. et al., 2025; Morris et al., 2022; Pan et al., 2022; Wang et al., 2026; Guo and Shao, 2025). Third, as mitochondrial damage progresses, mtDNA escapes via mPTP and VDAC1 channels, activating the cGAS-STING-TBK1-IRF3 pathway and NF-κB signaling, driving type I interferon responses and SASP establishment (Pan P. et al., 2025; Yang Y. et al., 2025; Zhao et al., 2023; Li et al., 2023; Wang M. et al., 2024; Zhou H. et al., 2025; Algieri et al., 2025; Xia et al., 2025). These three pathways collectively establish a self-amplifying inflammatory cascade that drives inflammaging. In addition, mitokines such as growth differentiation factor 15 (GDF15) released after activation of the mitochondrial unfolded protein response (UPRmt) further increase intestinal permeability by downregulating tight junction proteins, synergistically amplifying inflammatory signals with the aforementioned pathways (Ruiz-Malagón et al., 2025).

The inflammatory microenvironment further exacerbates gut dysbiosis by altering the intestinal oxygen gradient, inhibiting antimicrobial peptide secretion by intestinal epithelial cells, and promoting the overgrowth of facultative anaerobic pathobionts. The cycle is thus closed and self-amplified, ultimately driving the aging of systemic tissues and organs.

At the systemic level, the MMA vicious cycle reflects progressive complexity loss in the host-microbe symbiotic network—a concept supported by systems biology models of aging (Jazwinski and Kim, 2019). This study demonstrated that host and gut microbiome synchronously lose systemic complexity with biological age—manifested as declining microbiota alpha diversity—but not with chronological age, with UCP2/3 gene variations directly involved in aging network disintegration (Jazwinski and Kim, 2019). This explicitly positions the gut microbiota as an inherent component of the host aging regulatory network, rather than a passive bystander of time-related decline.

A recent study using a granulosa cell senescence model dissected the mtDNA leakage-cGAS-STING-cellular senescence axis (Pan P. et al., 2025). Immp2l deficiency-induced mitochondrial dysfunction upregulates TFAM, which paradoxically disrupts mtDNA nucleoid stability and promotes mtDNA escape via CypD-dependent inner membrane pores and VDAC1 channels. Cytoplasmic mtDNA activates cGAS-STING-TBK1-IRF3 signaling, upregulating SASP factors (IL-6, IL-8) and inducing inflammatory senescence. Impaired autophagosome-lysosome fusion further sustains this activation, highlighting synergy between defective mitophagy and innate immune signaling (Pan P. et al., 2025). This pathway provides direct experimental evidence for the causal cascade of mitochondrial damage-inflammation in the MMA vicious cycle.

**Figure 4:**
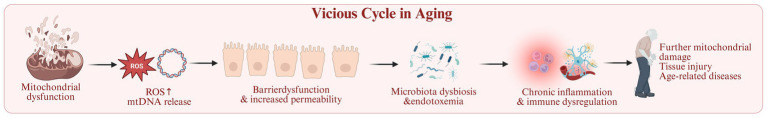
The self-reinforcing vicious cycle of the Microbiome-Mitochondria Axis in aging.

In summary, the vicious cycle centered on the dysregulation of bidirectional MMA communication and driven by both metabolic dysregulation and mitochondrial quality control failure is not a simple linear cascade reaction, but a multi-level, self-amplifying cellular network dynamic process with positive feedback as the core feature. Depletion of microbial metabolites impairs mitochondrial quality control capacity, while dysfunctional mitochondria reshape the host microenvironment through DAMP release and selectively restructure the gut microbiota. The complete closed loop formed by extracellular signal transduction, organelle response, microenvironment remodeling, and signal feedback constitutes the core cellular biological basis for the MMA driving the aging process. Within this closed loop, decreased mitophagy efficiency and sustained low-grade activation of the cGAS-STING pathway constitute two key signal amplification nodes, as well as the most promising targets for future intervention strategies (Figure 4).

Mitochondrial dysfunction triggers intestinal barrier damage, microbiota dysbiosis, and chronic inflammation via elevated mtROS, mtDNA leakage, and UPRmt-mediated pathways. These pathophysiological alterations further exacerbate mitochondrial damage, lead to tissue injury and age-related diseases, completing a self-reinforcing positive feedback loop that drives systemic aging.

### 4.2 The Microbiome-Mitochondria Axis as a cellular hub linking other hallmarks of aging

Rather than operating in isolation, the Microbiome-Mitochondria Axis is deeply embedded in the complex aging network, acting as a critical cellular hub that links upstream microbial signals to multiple classic hallmarks of aging downstream. At the systemic level, the deficit index analysis discussed above reveals that the host and gut microbiota synchronously lose complexity with increasing biological age, and this network disintegration phenomenon is closely associated with genetic variations in mitochondrial function-related genes (Jazwinski and Kim, 2019). This provides a macroscopic framework for understanding the association between this axis and the systemic aging process.

At the cellular level, one of the core effector functions of this axis is to reshape the host’s epigenetic landscape. Microbial metabolites, especially short-chain fatty acids (SCFAs), are the bridge linking this axis to epigenetic alterations. As comprehensively elaborated in existing reviews, SCFAs act as histone deacetylase inhibitors to regulate gene expression by relaxing chromatin structure (Owens et al., 2025; Borrego-Ruiz and Borrego, 2024). More in-depth, recent evidence details how the microbiome influences DNA and RNA methylation by regulating one-carbon metabolism and S-adenosylmethionine (SAM) availability (Rubas et al., 2025). These microbial-driven epigenetic changes directly affect aging-related gene expression programs. Clinical evidence further supports this link, with studies showing that the composition of an individual’s gut microbiota is directly correlated with accelerated epigenetic clocks based on DNA methylation, explicitly linking the state of this axis to the hallmark of epigenetic alterations (Torma et al., 2024).

Another key connection point of this axis is immunosenescence and chronic inflammation. Decreased gut microbiota richness has been confirmed to be associated with accelerated T cell senescence, especially in middle-aged and elderly populations (Madison et al., 2024). The immunosenescence framework also emphasizes the contributing role of the microbiome (Chen et al., 2025). Mechanistically, mtDNA leakage and activation of the cGAS-STING pathway caused by mitochondrial dysfunction are key drivers of sterile inflammation (Onyango et al., 2021). This pathway not only exacerbates the inflammatory cascade in local tissues, but also continuously reshapes the intestinal immune microenvironment, further amplifying the bidirectional positive feedback effect of gut dysbiosis-mitochondrial dysfunction-chronic inflammation at the systemic level, and serving as a key systemic driver of aging-related pathological progression (Jurcau et al., 2024). Notably, probiotic intervention can inhibit this inflammatory cycle by alleviating telomere attrition and enhancing mitochondrial function, thereby ameliorating aging-related cognitive decline (Zhou S. et al., 2025).

Furthermore, this axis exerts a profound impact on stem cell function. The evolutionary conservation of bacteria-mitochondria communication has been fully demonstrated (Lee et al., 2024), and recent studies have shown how this axis finely regulates the fate of intestinal stem cells (Andersson et al., 2025; Duan et al., 2024). One study found that asymmetric division of intestinal stem cells generates daughter cells enriched with old mitochondria, which drive TET-mediated epigenetic remodeling by producing more *α*-ketoglutarate, thereby promoting their differentiation into Paneth cells (Andersson et al., 2025). Another study revealed an exquisite regulatory layer: the intestinal epithelial gene Fut2 (whose expression is regulated by the microbiota) directly controls the efficiency of the mitochondrial respiratory chain and mitophagy by affecting the fucosylation of specific mitochondria-associated proteins, thereby determining the aging rate of intestinal stem cells (Duan et al., 2024). These findings collectively paint a picture in which the microbiome remotely modulates the mitochondrial quality and cell fate of stem cells through its metabolites or by influencing host gene modifications.

Notably, this axis is not only a passive reflection of the aging process, but also an actively intervenable regulatory node. A proof-of-principle study showed that the probiotic *Lactobacillus amylovorus KU4* inhibits the interaction between p53 and p300 by upregulating Necdin protein, thereby reducing p53 acetylation and transcriptional activity. This molecular event ultimately attenuated the senescence phenotype of adipocytes in aged mice, and improved mitochondrial function and systemic metabolic health (Yang G. et al., 2025). This finding directly confirms that microbial signals can remotely affect mitochondrial quality control and cellular senescence through the host protein regulatory network, providing feasibility evidence for anti-aging interventions targeting this axis.

We acknowledge that the MMA vicious cycle, as depicted, does not operate in isolation. Systemic drivers of aging—including progressive NAD^+^ depletion, mTORC1 hyperactivation, and global epigenetic drift—represent overarching upstream forces that could independently and concurrently drive both gut dysbiosis and mitochondrial deterioration (Yang L. et al., 2024; Jazwinski and Kim, 2019). Indeed, Jazwinski and Kim demonstrated that the host and gut microbiome synchronously lose systemic complexity with biological age, and genetic variations in core mitochondrial genes such as UCP2/3 are directly involved in the disintegration of this aging regulatory network, positioning the gut microbiota as an inherent but not exclusive component of the host aging network (Jazwinski and Kim, 2019). Furthermore, the MMA intersects with the hallmark of epigenetic alterations, as microbial metabolites modulate DNA/RNA methylation via one-carbon metabolism, while systemic epigenetic drift can independently reshape both mitochondrial gene expression and intestinal epithelial homeostasis (Rubas et al., 2025). Therefore, while we propose the MMA as a core local amplifier of aging, it should be viewed as an integral part of a larger hierarchical network, where it transduces and magnifies primary systemic metabolic and epigenetic insults into a self-sustaining organelle-microbiome loop. Disentangling these hierarchical relationships will be essential for precisely targeting the MMA without overriding beneficial systemic adaptations—a challenge we return to in Section 5.

In summary, as an integrative hub at the cellular level, the Microbiome-Mitochondria Axis links microbial signals to multiple hallmarks of aging, including genomic instability, telomere attrition, epigenetic alterations, mitochondrial dysfunction, cellular senescence, immunosenescence, and stem cell exhaustion. This deep interconnectivity means that perturbation of this axis by age, dietary, or environmental factors will trigger a cascade-amplified, self-reinforcing vicious cycle that accelerates systemic aging.

### 4.3 Age-related diseases as clinical manifestations of Microbiome-Mitochondria Axis failure

Chronic failure of the Microbiome-Mitochondria Axis is not only a driver of aging, but also a common pathophysiological basis for a series of seemingly unrelated age-related diseases. When the self-reinforcing cycle of this axis breaks through the body’s compensatory threshold, it manifests as organ-specific diseases.

Neurodegenerative diseases are a typical manifestation of axis failure in the central nervous system, often referred to as the collapse of the gut-brain-mitochondria axis. Comprehensive reviews have explicitly identified immunosenescence, mitochondrial dysfunction, and gut dysbiosis as the bridges linking aging to Alzheimer’s disease and Parkinson’s disease (Chen et al., 2025; Onyango et al., 2021). Age-related gut dysbiosis and impaired intestinal barrier function promote the entry of microbial-derived pro-inflammatory factors such as lipopolysaccharide (LPS) and harmful metabolites such as trimethylamine N-oxide (TMAO) into the circulation, exacerbating systemic and neuroinflammation (Jurcau et al., 2024). Postbiotics such as SCFAs and tryptophan derivatives have been shown to exert neuroprotective effects by regulating the gut-brain axis (Bashir et al., 2025). Direct evidence indicates that the microbial metabolite butyrate protects dopaminergic neurons from Parkinson’s disease-related toxicity by activating free fatty acid receptor 3 (FFAR3) (Getachew et al., 2020). Meanwhile, mitochondrial dysfunction in neurons and glial cells leads to energy depletion and oxidative stress, and mtDNA released from damaged mitochondria acts as a DAMP to further activate immune cells in the brain, forming an inflammaging microenvironment in the central nervous system. Probiotic intervention has been shown to improve cognitive function in senescence-accelerated mice by alleviating telomere attrition and enhancing mitochondrial bioenergetics (Zhou S. et al., 2025). The combined action of internal and external stress promotes the aggregation of pathological proteins such as amyloid-*β* (Aβ) and *α*-synuclein and neuronal death.

Cardiovascular diseases are a manifestation of axis failure in the circulatory system. The concept of “cardiovascular inflammaging” explicitly identifies gut dysbiosis as a key mechanism (Spray et al., 2025). Gut dysbiosis leads to a reduction in beneficial metabolites such as SCFAs, and an increase in harmful metabolites such as TMAO and LPS. In the context of atrial fibrillation, age-related mitochondrial dysfunction, telomere attrition, cellular senescence, and autophagy defects collectively drive atrial electrical and structural remodeling, while gut dysbiosis and its metabolites act as important aggravating factors (Wang M. F. et al., 2024). Systemic inflammation and metabolic disorders driven by axis failure ultimately lead to a series of clinical cardiovascular events, including hypertension, atherosclerosis, heart failure, and atrial fibrillation. Axis failure is not limited to the brain and heart. Idiopathic pulmonary fibrosis (IPF), a typical age-related lung disease, further exemplifies the synergistic effect of local microbiota dysregulation and mitochondrial dysfunction. A recent update reclassified IPF from the perspective of aging hallmarks, emphasizing that alterations in the lung and gut microbiota act in concert with impaired mitophagy to maintain the vicious cycle of inflammation and fibrosis (Torres-Machorro et al., 2025).

While these associations are compelling, they do not resolve the question of whether the Microbiome-Mitochondria Axis is a cause or consequence of age-related pathology. Beyond observational associations in sporadic diseases, monogenic progeroid syndromes provide strong genetic evidence for the causal role of this axis. Analysis of Fanconi anemia (FA) shows that defects in the nuclear DNA repair pathway are inextricably linked to secondary mitochondrial failure and mitophagy defects (Mejía-Barrera et al., 2026). Crucially, early signs of gut dysbiosis have been documented in FA patients, suggesting that primary mitochondrial fragility itself is sufficient to reshape the microbial landscape and trigger an inflammaging response (Mejía-Barrera et al., 2026). Therefore, this genetic model strongly validates that the Microbiome-Mitochondria Axis is a convergent downstream pathway in both pathological and physiological aging, rather than merely an epiphenomenon. This validation reinforces the view of this axis as a core pathway, whose dysfunction manifests as distinct diseases in different tissues according to local vulnerability.

Sarcopenia is a manifestation of axis failure in the skeletal muscle system. Chronic inflammation has been identified as a core driver, with the specific role of the gut-muscle axis discussed in detail (Liang and Zhang, 2026). Direct experimental evidence shows that the probiotic *Lactiplantibacillus plantarum* LM1001 improves muscle mass and strength in aged mice by regulating the gut microbiota, activating the IGF-1/Akt/FoxO3a signaling pathway, inhibiting the expression of muscle atrophy-related proteins such as MuRF1 and Atrogin-1, and promoting myogenic differentiation (Karekezi et al., 2025). Thus, the metabolic inflammatory storm and cellular energy crisis triggered by axis failure collectively lead to the progressive loss of muscle mass, strength, and function. Similarly, this protective effect extends to other metabolic tissues. As confirmed by the aforementioned study, targeted regulation of the gut microbiota can effectively ameliorate aging-related cellular senescence phenotypes and mitochondrial dysfunction, thereby improving the overall metabolic homeostasis of the body (Yang G. et al., 2025). Given the close association between adipose tissue aging and sarcopenia (i.e., sarcopenic obesity), this finding indicates that intervention strategies targeting the Microbiome-Mitochondria Axis have cross-organ systemic benefits, and can simultaneously improve the aging phenotypes of multiple metabolic organs.

Osteoarthritis (OA) is a direct manifestation of the failure of the Microbiome-Mitochondria Axis in joint tissues. The development of OA is closely related to multiple hallmarks of aging, especially cellular senescence and SASP driven by mitochondrial dysfunction, whose inflammatory factors and matrix-degrading enzymes directly disrupt cartilage homeostasis (Diekman and Loeser, 2024). Meanwhile, systemic low-grade inflammation caused by gut dysbiosis is also recognized as an important risk factor for OA. Therefore, OA can be regarded as the result of the combined action of local joint inflammaging and systemic metabolic inflammation.

Chronic gastritis and gastric cancer demonstrate the unique manifestation of axis failure in the gastrointestinal mucosa. The concept of “gastric inflammaging” has been proposed, suggesting that *H. pylori* infection, cellular senescence, mitochondrial dysfunction, and gut dysbiosis collectively form a self-reinforcing inflammatory microenvironment that drives the Correa cascade of chronic gastritis to atrophy, intestinal metaplasia, and even gastric cancer (Wang L. et al., 2025). This model not only deepens our understanding of the mechanism of gastric carcinogenesis, but also opens up new avenues for chemoprevention by targeting inflammaging.

In summary, major age-related diseases, including neurodegenerative diseases, cardiovascular diseases, idiopathic pulmonary fibrosis, sarcopenia, osteoarthritis, and gastric cancer, can all be regarded as clinical manifestations of the failure of the Microbiome-Mitochondria Axis in different organ systems. This perspective not only deepens our understanding of the common pathological basis of these diseases, but also provides a strong theoretical basis for the development of cross-disease therapeutic strategies targeting this axis.

## 5 Summary and future perspectives

### 5.1 Core conclusions

This review establishes the Microbiome-Mitochondria Axis (MMA) as a unified theoretical framework in which aging is driven by a self-reinforcing vicious cycle: gut dysbiosis depletes protective microbial metabolites (SCFAs, IPA, secondary bile acids), impairing mitochondrial quality control; dysfunctional mitochondria then trigger mtROS overproduction, immune metabolic reprogramming, mtDNA-cGAS-STING activation, and inflammaging, which in turn reshapes the gut microenvironment and perpetuates dysbiosis. The MMA thus functions as a cellular hub mechanistically linking epigenetic alterations, immunosenescence, and stem cell exhaustion, providing a unifying pathophysiological basis for age-related diseases across multiple organ systems.

### 5.2 Limitations

Several critical limitations warrant consideration: (1) most human studies are cross-sectional, precluding causal inference between gut dysbiosis and mitochondrial dysfunction; (2) confounding factors such as diet, medications, and lifestyle are often inadequately controlled; (3) methodological heterogeneity in microbiota profiling and mitochondrial assays complicates cross-study comparisons; (4) most mechanistic insights derive from animal models with uncertain translational validity to humans; and (5) no consensus exists on standardized biomarkers for assessing MMA integrity in clinical settings. Addressing these requires multidisciplinary efforts with standardized methodologies, longitudinal designs, and well-controlled intervention studies.

### 5.3 Priority future directions

We propose the following three priority directions for future research:

1. Establishing causality through mechanistic human studies. Controlled intervention studies—such as fecal microbiota transplantation (FMT) or targeted metabolite supplementation (butyrate, IPA) combined with longitudinal monitoring of mitochondrial function (e.g., PBMC respirometry, non-invasive MRS)—are urgently needed to determine whether restoring microbial metabolites directly improves mitochondrial bioenergetics in humans.
2. Developing integrated biomarkers of MMA health. Composite biomarkers integrating microbial metagenomic profiles, circulating metabolite panels (SCFAs, bile acids, tryptophan derivatives), and mitochondrial function indices (mtDNA copy number, plasma GDF15/FGF21) would enable early detection of axis dysregulation and provide surrogate endpoints for clinical trials.
3. Testing multi-target combination interventions. Given the self-reinforcing nature of the MMA vicious cycle, single-target approaches are unlikely sufficient. Promising strategies include probiotics/prebiotics (e.g., *L. amylovorus* KU4, *L. plantarum* LM1001), metabolite supplementation (butyrate, IPA, TGR5 agonists), and mitochondria-targeted agents (MitoQ, urolithin A, senolytics). Combining regimens that target both microbial and mitochondrial sides simultaneously, with careful evaluation of synergistic effects, represents the most logical next step.

In conclusion, the MMA is a biologically grounded, therapeutically actionable hub that integrates metabolism, immunity, and organelle quality control. Breaking this self-reinforcing cycle by restoring microbial metabolites, enhancing mitochondrial quality control, or dampening inflammatory amplification holds promise for systematically extending healthspan and requires interdisciplinary collaboration and rigorous clinical translation.
